# Spironolactone alleviates diabetic nephropathy through promoting autophagy in podocytes

**DOI:** 10.1007/s11255-019-02074-9

**Published:** 2019-02-08

**Authors:** Dan Dong, Ting-ting Fan, Ying-shi Ji, Jin-yu Yu, Shan Wu, Li Zhang

**Affiliations:** 1 Department of Nephrology, First Hospital of Jilin University, 71 Xinmin Street, Changchun, 130021 Jilin People’s Republic of China; 2 Basic Medical Science, Jilin University, Changchun, Jilin People’s Republic of China

**Keywords:** Diabetic nephropathy, Spironolactone, Podocytes, Autophagy, Rennin angiotensin aldosterone system

## Abstract

**Purpose:**

Podocytes are terminally differentiated cells lining the Bowman’s capsule. Podocytes are critical for the proper glomerular filtration barrier function. At the same time, autophagy is crucial for maintaining podocyte homeostasis and insufficient autophagy could cause podocyte loss and proteinuria that is commonly observed in diabetic nephropathy (DN).

**Methods:**

In this study, we investigated the role of spironolactone in podocyte loss and autophagy. DN model was established in male Sprague–Dawley rats using high-fat diet and low-dose streptozotocin. The impact of spironolactone on metabolic and biochemical parameters were tested by automatic biochemical analyzer. The angiotensin converting enzyme 1 and 2 (ACE1 and ACE2) and aldosterone were examined by ELISA. We examined the kidney histology and autophagy in podocytes by histochemical staining and electron microscopy. Podocyte loss and autophagy were analyzed by anti-NPHS2 and anti-WT1 as well as anti-Beclin1 and anti-LC3B, respectively.

**Results:**

Spironolacton decreased the urinary albumin excretion, lipids and fasting glucose levels, and alleviated kidney damage. Further, spironolactone increased the expression of the podocyte-specific markers WT1 and NPHS2, as well as the autophagic markers Beclin1 and LC3B (*P* < 0.05). Additionally, spironolactone partially blocked the rennin angiotensin aldosterone system (RAAS) by regulating the ACE1, ACE2 and aldosterone levels.

**Conclusions:**

In conclusion, spironolactone promoted autophagy in podocytes and further alleviated DN through partially blocking the RAAS.

## Introduction

DN is a devastating microvascular complication of diabetes mellitus (DM). Diabetic nephropathy (DN) accounts for up to 40% of end stage renal disease cases [1]. It is characterized by persistent albuminuria, impaired glomerular filtration rate and progressive decline in kidney function [2]. Podocytes are integral for the proper glomerular filtration barrier function. Podocytes are terminally differentiated cells lining the Bowman’s capsule that wrap around the capillaries [3]. Podocyte injury often results in massive proteinuria [4]. Since podocytes do not replicate, therefore, the intracellular degradation system is crucial for maintaining their homeostasis.

Autophagy, as a intracellular degradation system, is a conserved homeostatic process that is essential for maintaining cellular homeostasis under various conditions [5]. Previous studies demonstrated the importance of autophagy for the regulation of glucose and lipid metabolism in mammals. Impaired autophagy has been associated with the pathogenesis of several metabolic diseases [6–8]. Additionally, impairment of the autophagy was associated with severe podocyte injury and the pathogenesis of massive proteinuria due to DN [9].

Spironolactone, a non-selective mineralocorticoid receptor blocker, was observed to have beneficial role in streptozotocin (STZ)-induced diabetic rats [10]. Clinical trials also suggested that the addition of spironolactone to an angiotensin-converting enzyme inhibitor may further improve the outcome of proteinuria in patients with DN [11]. To this end, this study aimed to investigate whether spironolactone could alleviate podocyte loss via promoting autophagy in vivo and examined the underlying molecular mechanism of spironolactone function.

## Materials and methods

### Animals

A total of 85 male Sprague–Dawley rats (8 weeks old, weighing 200 ± 10 g) were individually housed in cages on a standard 12-h light/dark cycle with free access to food and water. Animals were randomly divided into a DN group (*n* = 55) and a control (CON) group (*n* = 30). In the DN group, rats were fed with high-fat diet for 1 month *ad libitum* followed by STZ injection (35 mg/kg; intraperitoneal injection) [12]. The high-fat diet (44.3 KJ/Kg) composition included 48% fat, 20% protein, 22% cholesterol, vitamins and microelements. STZ (Sigma, St. Louis, MO, USA) was dissolved in 0.1 mol/L citric acid buffer, pH 4.5. Before STZ injection, rats were allowed to fast for 12 h. Next, 3 days later, rats in the DN group were subjected to blood glucose analysis. Only rats with random blood glucose ≥ 16.7 mmol/L were regarded as successful DN model. Rats in the DN group were randomly divided into DN group (*n* = 15), DN + IN group treated with 2 units insulin twice a day (*n* = 15), DN + IN + SP group treated with 2 units insulin twice a day and spironolactone (40 mg/kg, dissolved in 3 mL water) once a day by gavage (*n* = 15). Rats in the control group were fed with a normal diet chow (25 KJ/Kg) that included 53% carbohydrates, 23% protein, 5% fat, vitamins and microelements *ad libitum* for 1 month. Next, those rats received intraperitoneal vehicle injections (0.1 mol/L citric acid buffer, pH 4.5) and were randomly divided into a CON group (*n* = 15) and a CON + SP group (*n* = 15). Rats in the CON + SP group were treated with spironolactone (40 mg/kg) once daily by gavage. Eight weeks later, all rats were anesthetized,and the kidneys were removed. A portion of the kidney tissue was fixed in 10% neutral formalin for histochemical analyses. A second portion of the kidney tissue was fixed in 2.5% glutaraldehyde for electron microscopy. Finally, segments of macroscopically and histologically identified renal cortex were obtained from the rat kidneys. Renal cortical tissues were passed through 100 µm and 200 µm meshes, and the podocyte rich residue (on the 200 µm mesh screen) was immediately frozen in liquid nitrogen and stored at − 80 °C until further analysis. The experimental protocol was revised and approved by the Basic Medical Science ethics committee of Jilin University. All measures were taken to decrease the animal discomfort.

### Biochemical parameters and ELISA

At the end of the experiment, blood samples were collected from the carotid artery, the sera were separated and analyzed by an automatic biochemical analyzer following the standard laboratory procedures. The examined biochemical parameters were fasting blood glucose (FBG), liver function tests including alkaline phosphatase (ALP), alanine transaminase (ALT), aspartate amino transferase (AST), and serum albumin(ALB). Kidney function tests included blood urea nitrogen (BUN) and serum creatinine (CRE). Blood lipid analysis included low-density lipoprotein cholesterol (LDL) and triglyceride (TG). Urine was collected in a metabolic cage for 24 h for urinary albumin excretion tests including 24-h microalbuminuria (mALB/24 h) and 24-h proteinuria (PRO/24 h) with pyrophenol red molybdate staining. All biochemical analyses were performed at the Department of Laboratory Medicine, the First Hospital of Jilin University.

Alternatively, blood samples (3 mL) were rapidly injected into an EDTA vacuum anticoagulant tube or vacuum coagulation tube and the plasma was separated. Plasma samples were used for the detection of angiotensin-converting enzyme 1 and 2 (ACE1 and ACE2), as well as aldosterone detection by ELISA kits (R&D systems, Minneapolis, MINN, USA). Optical density was measured at 450 nm.

### Western blotting

Kidney tissues (40 µg) were homogenized in 1 mL of RIPA Buffer (Solarbio, R0020, Beijing, China) containing 10 µL of phenyl methyl sulfonyl fluoride as a protease inhibitor. Next, proteins (60 µg) were separated on a 10% SDS-polyacrylamide gel electrophoresis and transferred onto a polyvinylidene fluoride membrane. Membranes were blocked with casein and subsequently incubated with the following primary antibodies at 4 °C overnight: anti-Wilms Tumor (anti-WT1, 1:1000, Abcam, USA), anti-Nephrosis2 (anti-NPHS2, 1:1000, Abcam, USA), rabbit anti-Beclin1 (1:1000, Abcam, USA), rabbit anti-LC3B (1:1000, Abcam, USA). Next, membranes were probed with horseradish peroxidase-conjugated goat anti-mouse IgG or goat anti-rabbit IgG for 1 h at room temperature (Beyotime, Beijing, China). Following washing, membranes were visualized using High-sig ECL Western Blotting Substrate (Tanon, Shanghai, China) and the protein bands were analyzed using densitometry with Image J.

### Real-time PCR

RNA was extracted from the renal tissues using Ultrapure total RNA rapid extraction kit (Bioteke, RP1201, Beijing, China). RNA samples were reverse-transcribed using 5× All-in-One RT MasterMix (Applied Biological Materials Inc., G485&G486&490, Vancouver, Canada) in a 10 µL volume. Next, we performed real-time PCR using EvaGreen 2X qPCR MasterMix- Low ROX (Applied Biological Materials Inc., Vancouver, Canada) in a 20 µL reaction volume. Primers used for quantification were Beclin 1 forward:5′-TGCAGGTGAGCTTCGTGTG-3′ and reverse:5′-CTGGGCTGTGGTAAGTAATGGAG-3′; GAPDH forward:5′-CAAGTTCAACGGCACAGTCAA-3′ and reverse:5′- TGGTGAAGACGCCAGTAGACTC-3′. Relative mRNA levels were determined using the comparative threshold cycle method followed by normalization to the GAPDH mRNA level. Each experiment was performed in duplicates and repeated three times. The used real-time PCR protocol was: 95 °C for 10 min, 40 cycles of 95 °C for 15 s followed by 60 °C for 1 min, 95 °C for 15 s, 60 °C for 30 s, and 95 °C for 15 s.

### Histochemical staining

Paraffin sections were de-waxed and rehydrated in a descending ethanol series. Sections were stained with HE or PAS staining according to the standard protocols.

### Electron microscopy

Renal tissue was fixed in 2.5% glutaraldehyde for 2 h, then fixed in 10% osmium tetroxide for another 2 h and embedded in Epon812 resin. After ultra-cutting, sections were stained with lead citrate and the kidney ultrastructure was observed under electron microscope (FEI, Hillsboro, Oregon, America) at 10,000× or 5000×.

### Statistical analysis

Results were expressed as mean ± SEM. All experiments were repeated at least three times. All statistical analyses were performed using SPSS version 20.0 (SPSS Inc., Chicago, IL). ANOVA was used for the comparison between groups. A *P* < 0.05 was considered to be statistically significant.

## Results

### Spironolactone lowered lipid and glucose levels and improved the liver and kidney functions

We assessed the liver and kidney functions, blood lipid, FBG, kidney to body weight ratio (KW/BW), urinary albumin excretion among the examined groups (Table 1). Compared to the CON group, the levels of ALP, ALT, BUN, FBG, LDL, TG, TP, KW/DW, mALB/24 h levels were significantly increased in the DN group (*P* < 0.05; Table 1). On the other hand, the CRE level was significantly decreased (*P* < 0.0001). Additionally, ALB level was decreased while AST and PRO/24 h levels were increased in the DN group, but they did not achieve statistical significance (*P* > 0.05). Insulin treatment for 8 weeks resulted in the significant increase of ALB level and the decrease of ALP, BUN, KW/BW, mALB/24 h in the DN + IN group (*P* < 0.05; Table 1). Combined spironolactone and insulin treatment further improved ALB levels and decreased the BUN, ALP, KW/DW, mALB/24 h, LDL, TG and GLU levels in the DN + IN + SP group (*P* < 0.05; Table 1). Taken together, our results demonstrated that spironolactone and insulin treatment lowered the lipid and glucose levels, improved the liver and kidney function as well as decreased the urinary albumin excretion compared to insulin treatment alone (Table 1).

**Table 1:** Metabolic and biochemical parameters among the different experimental groups

	DN	DN + IN	DN + IN + SP	CON + SP	CON
ALB (g/L)	21.64 ± 1.21	23.92 ± 2.38*	23.87 ± 1.63*	25.74 ± 1.92^#^	23.07 ± 1.59
ALP (U/L)	783.26 ± 147.07	407.85 ± 153.76*	287.45 ± 84.26*	149.28 ± 42.29	184.32 ± 54.73*
ALT (U/L)	268.33 ± 205.34	168.03 ± 110.05	90.48 ± 13.51*	90.54 ± 38.25	77.37 ± 9.30*
AST (U/L)	306.94 ± 215.73	256.42 ± 252.65	134.38 ± 20.56	144.80 ± 8.51^#^	159.68 ± 5.95
BUN (mmol/L)	12.92 ± 2.81	9.85 ± 1.07*	9.74 ± 1.51*	8.19 ± 0.83	8.20 ± 0.32*
CHO (mmol/L)^$^	2.03 ± 0.38	2.06 ± 0.31	1.86 ± 0.17*	1.38 ± 0.33^#^	1.80 ± 0.19
CRE (umol/L)	26.27 ± 2.13	25.57 ± 4.31	28.17 ± 2.28	33.76 ± 4.32	35.92 ± 3.40*
GLU (mmol/L)	38.39 ± 1.50	31.96 ± 8.60	26.81 ± 5.96*	10.86 ± 2.16^#^	16.21 ± 1.97*
LDL(mmol/L)^$^	0.43 ± 0.07	0.37 ± 0.07	0.30 ± 0.02*	0.28 ± 0.08^#^	0.35 ± 0.05*
TG (mmol/L)^$^	2.67 ± 1.33	2.48 ± 1.29	1.03 ± 0.22*	0.57 ± 0.08^#^	0.99 ± 0.20*
TP (g/L)	60.86 ± 3.30	61.97 ± 3.19	61.42 ± 3.86	65.06 ± 4.15^#^	56.08 ± 2.29*
KW/BW	5.84 ± 70.44	4.18 ± 40.95*	3.65 ± 7.58*	3.13 ± 28.57	3.19 ± 18.81*
PRO/24 h (g)	0.22 ± 0.2	0.06 ± 0.04	0.05 ± 0.01	0.02 ± 0.01	0.04 ± 0.02
mALB/24 h (mg)	4.73 ± 2.02	1.46 ± 1.63*	1.12 ± 0.98*	0.25 ± 0.13	0.41 ± 0.14*

Data are mean ± SEM. *CON,DN + IN and DN + IN + SP VS. DN, *P* < 0.05. ^#^CON VS.CON + SP, *P* < 0.05. ^$^DN + IN VS. DN + IN + SP, *P* < 0.05

### Spironolactone alleviated kidney injury

**Fig. 1:**
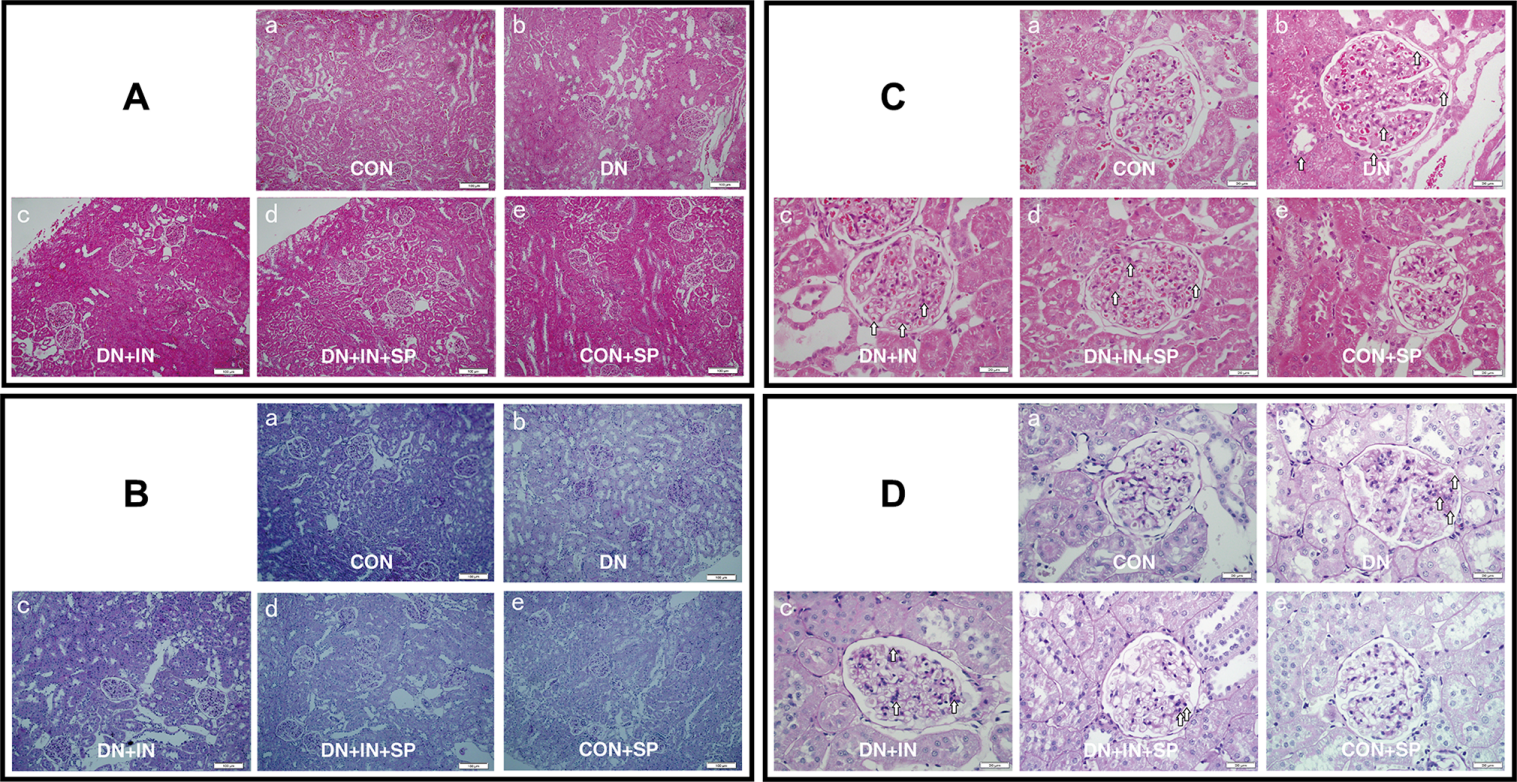
Spironolactone alleviated kidney damage in DN rats. **a, c** Representative HE staining of renal tissues from the different groups. **a** Magnification, ×100. **c** Magnification, ×400. The white arrows define the lesion location. **b, d** Representative PAS staining of renal tissues from the different groups. **b** Magnification, ×100. **d** Magnification, ×400. The white arrows define the lesion location. **Aa**–**Da** In the CON group and **Ab**–**Db** in the CON + SP group, normal glomerulus were seen under a light microscope. Glomerular blood vessels are thin and clear. The number of endothelial cells and mesangial cells are normal. The surrounding renal tubules are also normal; **Ac**–**Dc** In the DN group, the glomerular basement membranes were slightly thickened, the mesangial matrix showed segmental hyperplasia and the renal tubular epithelial cells were presented with vacuolar degeneration, but no atrophy was observed. Similarly, there was no fibrosis in the renal interstitial of the DN group; **Ad**–**Dd** In the DN + IN group, the glomerular basement membranes were still slightly thickened and the mesangial matrix showed segmental hyperplasia; but, overall, the observed histological changes were less severe than that of the DN group. **Ae**–**De** In the DN + IN + SP group, the glomerular basement membranes were slightly thickened, and the mesangial matrix was mildly hyperplastic. No abnormalities were observed in the renal tubules and renal interstitial

Next, we used HE and PAS staining to examine the kidney tissues and study the impact of spironolactone treatment (Fig. 1a–d, respectively). PAS staining is mainly used to observe the change of basement membrane. In the CON and CON + SP groups, glomerular blood vessels are thin and clear (Fig. 1a–d). The number of endothelial cells and mesangial cells and the surrounding renal tubules are normal. In the DN group, the glomerular basement membranes were slightly thickened, the mesangial matrix showed segmental hyperplasia and the renal tubular epithelial cells were presented with vacuolar degeneration, but no atrophy was observed (Fig. 1Ab–Db). Similarly, there was no fibrosis in the renal interstitial of the DN group. In the DN + IN group, the glomerular basement membranes were still slightly thickened and the mesangial matrix showed segmental hyperplasia; but, overall, the observed histological changes were less severe than that of the DN group (Fig. 1Ac–Dc). Following insulin and spironolactone treatment (DN + INS + SP group) the glomerular basement membranes were slightly thickened, and the mesangial matrix was mildly hyperplastic than those of the DN + IN group (Fig. 1Ad–Dd). No abnormalities were observed in the renal tubules and renal interstitial. Taken together, spironolactone alleviated kidney injury induced by STZ and high-fat diet in the DN model.

**Fig. 2:**
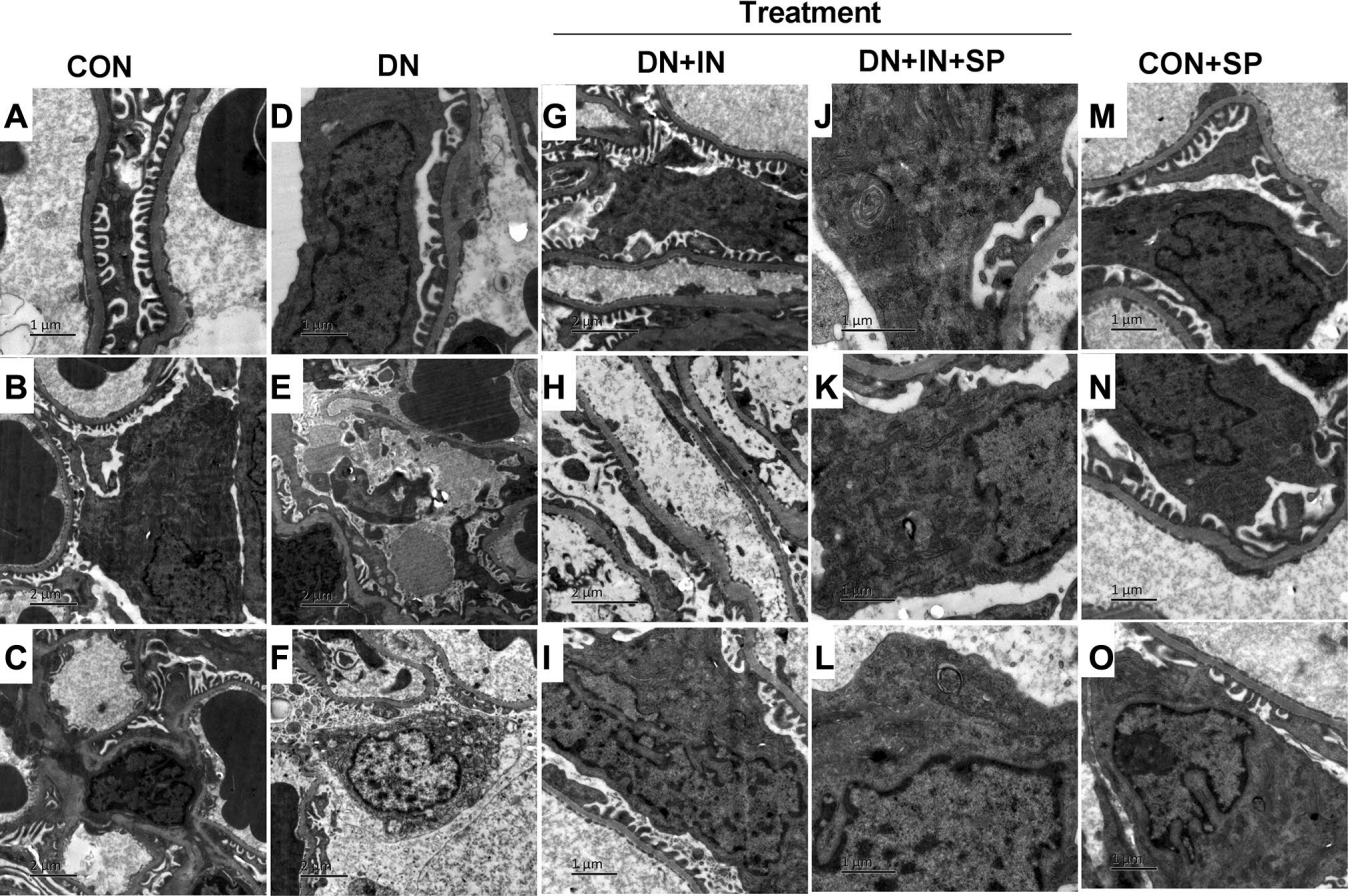
Spironolactone alleviated ultrastructural damage in DN renal. Electron microscopy micrographs representing ultrastructural changes in the renal tissues to observe basement membranes, mesangial matrix, podocyte foot processes, mitochondrial and autophagy in podocytes. Magnifcation, ×10,000 or ×5000. **a** The basement membrane was uniform in thickness and clear in structure, and the structure of the foot process was normal. **b** Podocytes were bulky, had slightly oval core, and located on the side of the cell. Mitochondria, rough endoplasmic reticulum, microtubules, and microfilaments were observed in the cytoplasm. **c** A mesangial cell was seen in the mesangial area, with a star shape and a large nucleus. **d** Partial foot process fusion, the mesangial matrix was inserted into the basement membrane to form a double-track sign. **e** Podocyte nucleus pyknosis, chromatic condensation, convergence of mitochondria in podocyte protrusion, fusion of foot processes, and an uneven thickness of basement membrane were observed. **f** The podocyte local cell membrane cytoplasm cavitation, mitochondrial cavitation, hernia fracture disappears, rough endoplasmic reticulum expansion, and uneven thickness of basement membrane were observed. **g, h** Uneven thickness of basement membrane and foot process local fusion were observed. **i** The podocyte was bulky. The nucleus was irregularly shaped. The mitochondria showed signs of slight cavitation. The limulus structure was unclear, and the rough endoplasmic reticulum had no obvious expansion and change, showing more Golgi complexes. **j** The podocyte structure was almost normal and concentric bodies were visible in the cytoplasm. **k** Normal mitochondria and rough endoplasmic reticulum in podocyte, partially myeloid body structure was visible. **l** The structure of mitochondria and rough endoplasmic reticulum in podocytes was normal. More Golgi complexes were seen, and there was an autophagosomal body near the cell membrane. **m** The basement membrane was uniform in thickness and clear in structure. The structure of the podocyte was normal, and fusion of the foot processes was not seen. **n** The basement membrane was uniform in thickness and clear in structure. The structure of the podocyte was normal, and fusion of the foot processes was not seen. **o** The basement membrane was uniform in thickness and clear in structure. The structure of mitochondria and rough endoplasmic reticulum in the podocyte was normal, and the lysosome and myeloid body structure were seen. Fusion of the foot process was not observed

The impact of spironolactone on the urinary albumin excretion and the glomerular basement membrane led us to use electron microscopy to investigate the podocyte damage caused by DN in more details (Fig. 2). In the CON group, The basement membrane was uniform in thickness and clear in structure, and the structure of the foot process was normal. The mesangial matrix did not show signs of hyperplasia (Fig. 2a–c). In the DN group, the glomerular basement membranes were unevenly thickened, the mesangial matrix appeared like a double orbit sign. Further, signs of podocyte damage were evident including nuclear pyknosis, chromatic condensation, the podocyte cytoplasm cavitation, mitochondrial cavitation, hernia fracture disappeared, and rough endoplasmic reticulum expansion and fusion of foot processes (Fig. 2d–f). Following insulin treatment, uneven thickness of basement membrane was also observed as well as podocyte damage. Podocyte foot processes demonstrated local fusion and mild mitochondrial cavitation was also observed (Fig. 2g–i). Following insulin and spironolactone treatment (DN + INS + SP group), podocyte morphology regained a relatively normal appearance and autophagic vacuoles were frequently observed in podocytes. The podocyte foot processes were presented with mild diffusion (Fig. 2j–l). In the CON + SP group, podocyte morphology was normal similar to the CON group (Fig. 2m–o). In conclusion, spironolactone treatment alleviated podocyte damage among rats in the DN group.

### Spironolactone enhanced autophagy in podocytes and alleviated podocyte loss

**Fig. 3:**
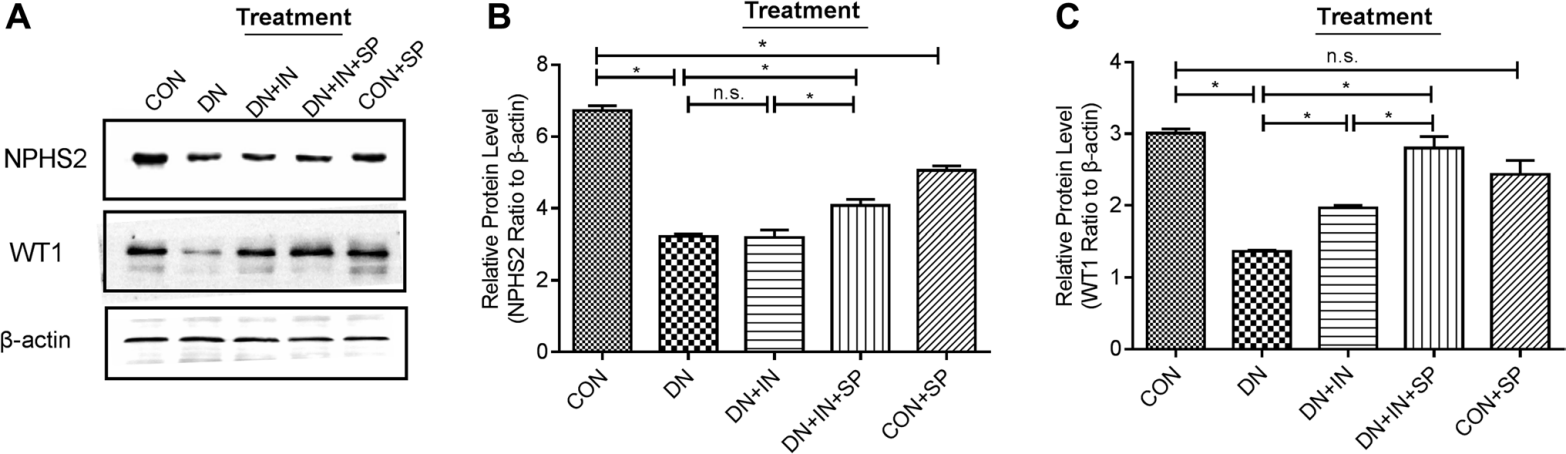
Spironolactone alleviated podocyte loss in DN rats. **a** Expression of protein levels of NPHS2 and WT1; renal lysates (50 µg) were immunoblotted with anti-NPHS2, anti-WT1. Tublin was used as a loading control. **b, c** The relative densities of each band were analyzed and normalized to the Tublin level. The experiments were repeated three times. Values are expressed as means ± SEM of each group relative to Tublin. *P < 0.05. *n.s* no statistical significance

We analyzed specific immunohistochemical markers for podocytes (WT1 and NPHS2) using western blotting [13]. In the DN group, we observed that the expression of WT1 and NPHS2 were significantly lower in the DN group compared to the CON group (*P* < 0.001; Fig. 3a–c). After insulin and spironolactone treatment, the expression of WT1 (*P* < 0.001) and NPHS2 (*P* = 0.009) in DN + IN + SP group was significantly higher than that of the DN + IN group (Fig. 3a–c). These results suggest that spironolactone treatment alleviated podocyte loss in the DN group.

**Fig. 4:**
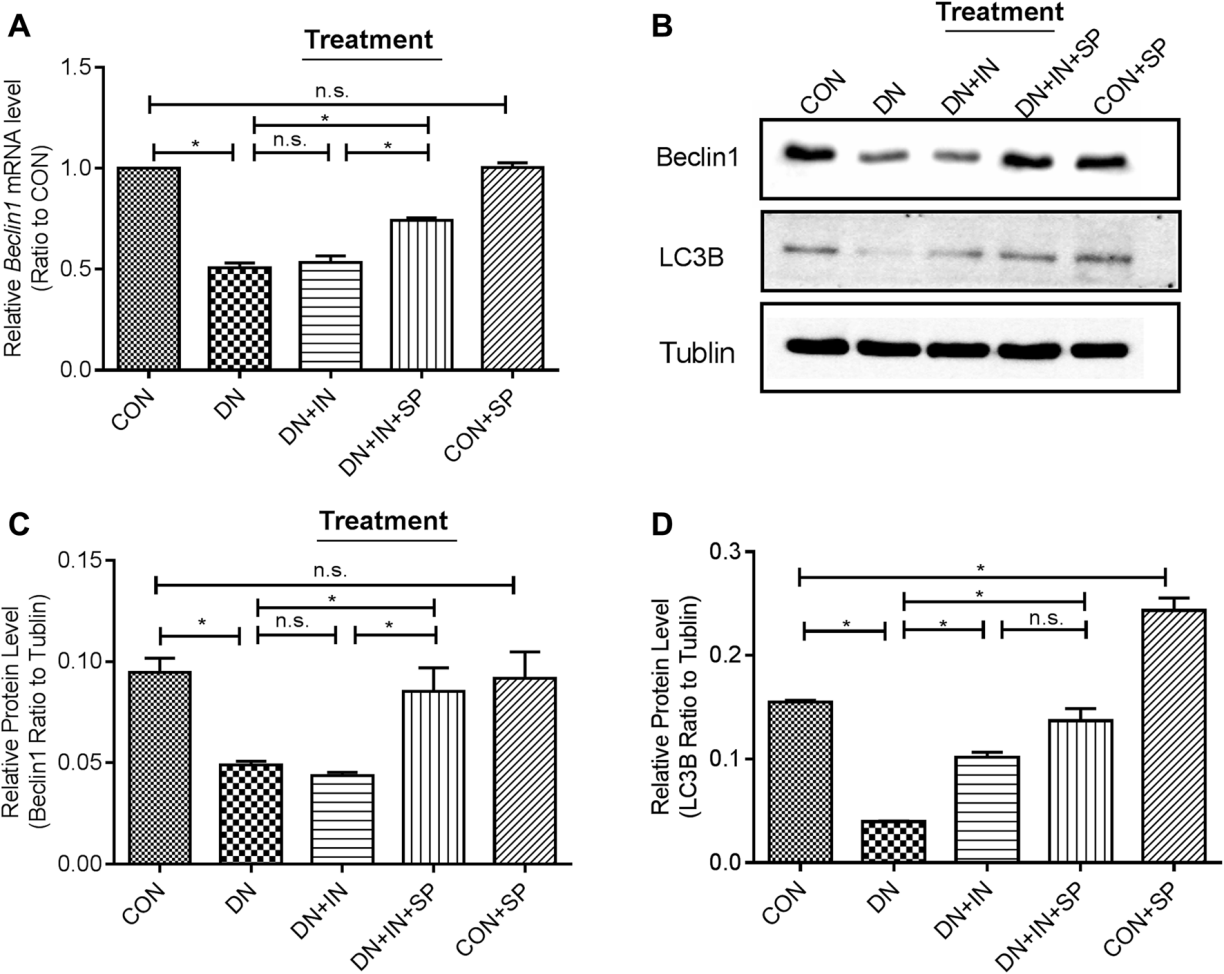
Spironolactone alleviated autophagy in podocytes. **a** Expression of Beclin-1 mRNA by RT-PCR. The experiments were repeated three times. The values are expressed as means ± SEM of each group normalized to GAPDH, and then relative to CON. **b** Expression of protein levels of Beclin1 and LC3B; renal lysates (50 µg) were immunoblotted with anti-Beclin1, anti-LC3B antibodies. β-actin was used as a loading control. **c, d** The relative densities of each band were analyzed and normalized to the β-actin level. Values are expressed as means ± SEM. *P < 0.05. *n.s* no statistic significance

Previous studies demonstrated that the impairment of autophagy-lysosome system was associated with severe podocyte damage [9]. Therefore, we examined the effect spironolactone on autophagy by analyzing the expression of the autophagic markers, Beclin1 and LC3B [14] (Fig. 4). Western blotting demonstrated that the expressions of Beclin1 and LC3B were significantly lower in the DN group than in the CON group (*P* < 0.05; Fig. 4b–d). Similarly, the expression of Beclin1 mRNA was significantly lower in the DN group (*P* < 0.0001, Fig. 4a). Following, insulin and spironolactone treatment, the expression of Beclin1 and LC3B was significantly increased (*P* < 0.05; Fig. 4b–d). Likewise, Beclin1 mRNA level was significantly upregulated in the DN + IN + SP group (*P* = 0.0007; Fig. 4a). These results imply that spironolactone alleviated podocyte loss by promoting autophagy.

In the CON + SP group, the expression of LC3B was significantly higher (*P* = 0.002; Fig. 4d), but the expression of NPHS2 (*P* < 0.001; Fig. 3b) was lower compared to the CON group. This result implies that spironolactone may have a side effect on normal foot cells.

### Spironolactone treatment partially blocked the rennin angiotensin aldosterone system (RAAS)

**Fig. 5:**
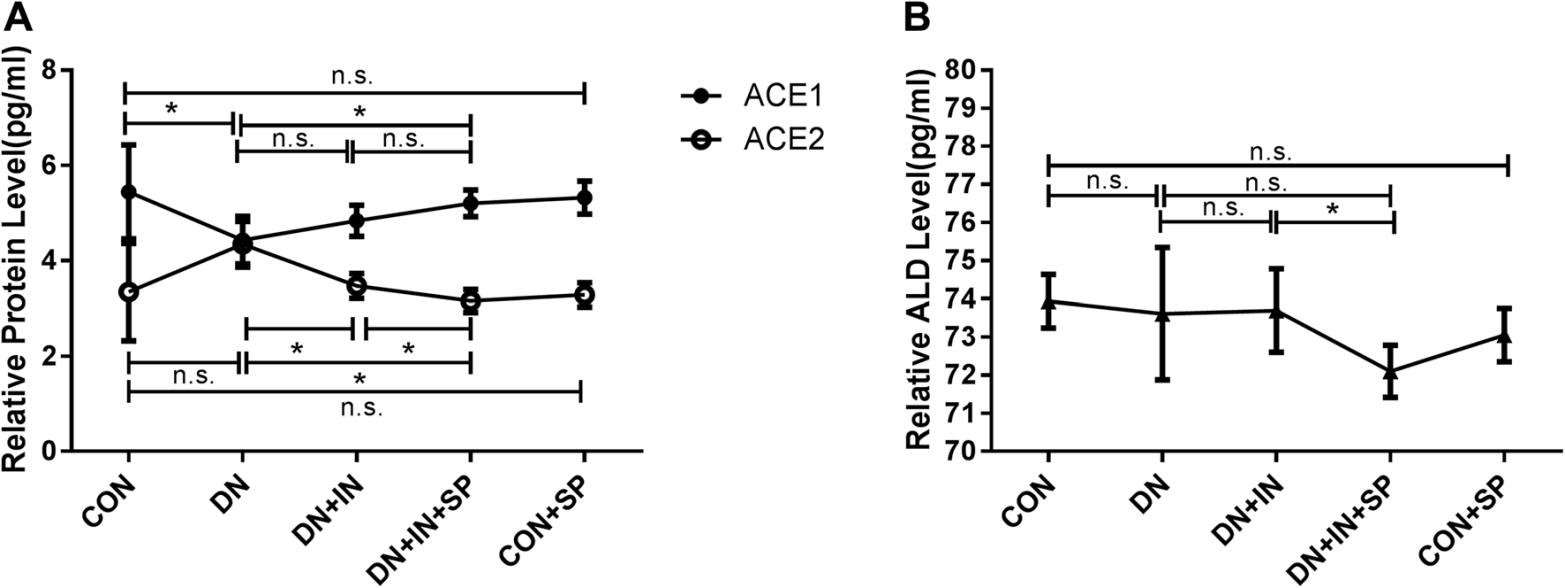
Spironolactone partially blocked the RAAS. Plasma ACE1, ACE2, and aldosterone were quantified by ELISA using rat ACE1, ACE2 and ALD ELISA kit. **a** Qquantitation of serum ACE1 and ACE2. **b** Quantitation of serum aldosterone. The values are expressed as means ± SEM of each group. *P < 0.05. *n.s*. no statistical significance

To gain insights regarding the underlying mechanism for spironolactone function, we analyzed the influence of spironolactone on RAAS. Plasma ACE1, ACE2 and aldosterone levels were quantified by ELISA (Fig. 5). In the DN group, the ACE2 level was significantly higher (*P* = 0.042; Fig. 5a); while, ACE1 level was significantly lower than that of the CON group (*P* = 0.037; Fig. 5a). The aldosterone level was not significantly different between the two groups (*P* = 0.674; Fig. 5b). Compared to the DN group, the ACE1 level was not significantly different after insulin treatment in the DN + IN group (*P* = 0.121; Fig. 5a), but it was upregulated after insulin and spironolactone treatment (*P* = 0.007; Fig. 5a).Additionally, upon insulin or insulin and spironolactone treatment, ACE2 level was significantly downregulated in the DN + IN and the DN + IN + SP groups (*P* = 0.0002; Fig. 5a). Following insulin and spironolactone treatment, the aldosterone level was significantly downregulated (*P* = 0.037, Fig. 5b). In conclusion, spironolactone treatment could partially block the RAAS by regulating the levels of ACE1, ACE2 and aldosterone.

## Discussion

Autophagy is conserved catabolic mechanism by which cytoplasmic components are transported to the lysosomes for degradation [15]. In this study, our results demonstrated that impaired autophagy in podocytes could possibly contribute to the pathogenesis of podocyte loss which will ultimately result in proteinuria and hence DN development. Interestingly, spironolactone treatment alleviated podocyte loss through partially blocking the RAAS system and promoting autophagy in podocytes.

In this study, high-fat diet feeding along with low STZ dose resulted in the development of DN. Compared to other models, the high-fat diet + low-dose STZ model has great similarity to the human type 2 diabetes and is easier to establish [16]. We found that spironolactone treatment improved the liver and kidney function and decreased the urinary albumin excretion. Moreover, spironolactone lowered the elevated blood glucose and lipids levels compared to insulin monotherapy.

Our results indicated the podocyte loss in DN model. Accumulating evidence indicates that autophagy plays a critical role in the maintenance of the kidney tissues against diseases and aging [17]. For instance, autophagy was reported to be activated in doxorubicin-induced nephropathy [18] to protect against podocyte injury [19]. In *Podo-Atg7*, podocyte-specific knock out mice, doxorubicin treatment resulted in podocyte injury, glomerulopathy, and proteinuria [20]. Autophagy deficiency in the proximal tubule was associated with higher susceptibility for ischemic damage and promotesrenal fibrosis [21]. Moreover, Tagawa et al. reported that impaired podocyte autophagy exacerbates proteinuria in diabetic nephropathy [22]. Indeed, here we demonstrated the insufficiency of autophagy in podocytes of DN rats. Therefore, it is plausible to speculate that insufficient podocyte autophagy plays a pathogenic role in proteinuria development.

Spironolactone is a first-generation mineralocorticoid receptor antagonists (MRAs). spironolactone treatment is recommended as antihypertensive treatment and it reduces blood pressure and may offer additional renoprotection in type 1 diabetic patients with diabetic nephropathy [23]. Han et al. previously demonstrated that spironolactone prevents diabetic nephropathy through an anti-inflammatory mechanism in type 2 diabetic rats [24]. Randomized studies confirmed that the addition of MRAs to a renin–angiotensin system (RAS) blocker reduced albuminuria resulting from diabetic or nondiabetic causes [25, 26]. Additionally, the use of spironolactone alone was similarly effective to the use of spironolactone and losartan combination in reducing albuminuria [27]. In combination with, conventional RAS inhibitors, spironolactone could successfully reduce albuminuria in patients with diabetic nephropathy [28]. In vitro spironolactone was observed to decrease podocyte motility under high-glucose conditions [29]. Additionally, spironolactone promotes autophagy via inhibiting PI3K/AKT/mTOR signaling pathway and reduce the adhesive capacity damage in podocytes under mechanical stress [30]. Therefore, spironolactone was proposed to promote the podocyte autophagy in DN; thereby, reducing podocyte damage and thus it can be used as an effective drug for diabetic nephropathy [31]. Indeed, our results indicated that treatment with spironolactone decreased urinary albumin excretion, and reduced the podocytes loss as well as enhanced the histological score of the renal tissues. Additionally, spironolactone enhanced autophagy of podocytes in the DN rats, which came in agreement with previous in vitro studies [32]. To the best of our knowledge, this is the first study that demonstrates the beneficial effects of spironolactone on the podocyte autophagy in an in vivo DN model.

RAAS is a major regulator of blood pressure control, fluid, and electrolyte balance in humans [33]. Angiotensin II (Ang II), an important component of RAAS, could promote autophagy in podocytes [34]. ACE1 generates the physiologically active peptide Ang II by cleaving the C terminal dipeptide His–Leu from Ang I [35]. ACE2 is associated with the degradation of Ang II to Ang-(1–7) and Ang I to Ang-(1–9) [36, 37]. In this study, our results demonstrated the downregulation of ACE1 and the upregulation of ACE2 in DN rats. This could lead to lower Ang II levels and attenuated autophagy in podocytes. Following spironolactone treatment, ACE1 was upregulated and ACE2 was downregulated in the DN + INS + SP group. Accordingly, we speculated that the upregulation of ACE1 would increase the Ang II synthesis and downregulation of ACE2 will decrease the Ang II degradation. Our results showed that sub-normal levels of AngII could attenuate podocyte autophagy in DN. Therefore, it is reasonable to speculate that spironolactone promoted autophagy in podocytes via the upregulation Ang II nevertheless, previous studies proposed that high Ang II levels could promote apoptosis and induce oxidative stress [38]. This discrepancy could be attributed to the importance of maintaining Ang II or RAAS homeostasis. However, future studies are required to confirm our results and further explore the underlying molecular mechanism.

Aldosterone promotes glomerular and tubular-interstitial inflammation and fibrosis through various pathways causing chronic kidney diseases and albuminuria [39]. The use of ACEI or angiotensin receptor blockers improves the renal outcomes in patients with chronic kidney diseases through blocking the RAAS [40]. However, the long-term use of those treatments is hampered by aldosterone escape [41]. In this study, we observed that spironolactone treatment decreased the aldosterone level in the CON and DN groups. Therefore, spironolactone could at least partially block the RAAS system and aldosterone escape.

## Conclusion

In conclusion, here, our results demonstrated that spironolactone promoted autophagy in podocytes and further alleviated DN by partially blocking the RAAS. Results obtained here show that spironolactone could be instrumental for the treatment of DN.
